# Low-Level Laser Therapy with a 635 nm Diode Laser Affects Orthodontic Mini-Implants Stability: A Randomized Clinical Split-Mouth Trial

**DOI:** 10.3390/jcm9010112

**Published:** 2019-12-31

**Authors:** Rafał Flieger, Tomasz Gedrange, Kinga Grzech-Leśniak, Marzena Dominiak, Jacek Matys

**Affiliations:** 1 Private Dental Practice, 64-000 Kościan, Poland; gabinet6@op.pl; 2 Dental Surgery Department, Medical University of Wroclaw, 50-425 Wroclaw, Poland; Tomasz.Gedrange@uniklinikum-dresden.de (T.G.); marzena.dominiak@wp.pl (M.D.); 3 Laser Laboratory at Dental Surgery Department, Medical University of Wroclaw, 50-425 Wroclaw, Poland; kgl@periocare.pl

**Keywords:** ATP, biostimulation, micro-screw, periotest, semiconductor laser

## Abstract

Background: The study aimed to clinically estimate an influence of a 635 nm diode laser on the stability of orthodontic mini-implants, to assess mini-implants loss, and to evaluate a pain level after the treatment. Materials and Methods: The randomized clinical split-mouth trial included 20 subjects (13 women and 7 men; age: 32.5 ± 6.1 years), 40 implants (RMO, West Colfax Ave., Denver, CO, USA) with a diameter 1.4 mm and length of 10 mm. Mini-implants were placed in the area of the attached gingiva between the second premolar and first molar teeth, 2 mm below the mucogingival junction of both sides of the maxilla. Each implant on the right side (G1, *n* = 20) of the maxilla was irradiated with a diode laser, and the implants on the opposite side (left, G2, *n* = 20) were a control group (without laser irradiation). The 635-nm laser parameters; dose: 10 J per point (20 J/cm^2^), time: 100 s per point, two points (irradiation on a buccal, and a palatal side of the alveolus/implant), the total energy per session 20 J. Laser application protocol: immediately and 3, 6, 9, 12, 15, and 30 days after surgery. The total energy after all therapeutic sessions was 140 J. The implants’ stability was measured employing a Periotest device (Periotest Test Value—PTV) immediately and 3, 6, 9, 12, 15, 30, and 60 days after the insertion of the mini-implants. Results: We found significantly higher secondary stability, lower mean PTV (6.18 ± 5.30) and (1.51 ± 2.25), for self-drilling mini-implants (G1, test group) in contrast to the control, G2 group (9.17 ± 8.25) and (5.00 ± 3.24), after 30 (*p* = 0.0003) and 60 days (*p* = 0.0000). Moreover, the analysis of the mini-implants stability after 635-nm diode laser application revealed significant higher stability in comparison with none irradiated implants (G2 group) after 3 days. (*p* = 0.0000) There was no significant difference in pain level measured on the NRS-11 scale on both sides of the maxilla. (*p* = 0.3665) An important finding was that all inserted mini-implants survived during a two-month observation period. Conclusions: 635-nm diode laser at laser irradiation increases the secondary stability of orthodontic mini-implants.

## 1. Introduction

Orthodontic microimplants are increasingly used in orthodontic treatment [1]. Thanks to the use of microimplants, clinicians have managed to overcome such problems as instability of anchoring or dependence on compliance with regulations, which occur in the case of the traditional anchoring method [2]. However, according to Beak et al. [3], the losses of microimplant usually occur during the first weeks after loading. Therefore, improving early stabilization is an important step towards increasing the reliability of mini-implant therapy [4]. The primary stabilization of the mini implants is achieved through mechanical retention. Mini-implants can be loaded immediately after the treatment. However, it is not necessary to maintain a specific time of osseointegration or tissue healing. [5]

The volume and quality of the bone that affect the osseointegration process are the most critical factors responsible for the long-term clinical success in implant dentistry [6]. Moreover, sufficient primary stability of mini-implants is a crucial factor enabling their immediate or early loading [5]. The stability of the mini-implants is determined as biomechanical stability upon implant placement [5]. It depends on bone form at the bone–implant interface [5,6,7]. The level of obtained implants stability includes bone quality and volume, mini-implant surface morphology, and surgical method [8].

In implant dentistry and mini-implant assisted orthodontics, a sufficient process of measuring of implant’s stability, and bone density is essential [6]. Because the removal torque method and histomorphometric analysis measurements are invasive techniques [9], Periotest and resonance frequency analysis (RFA) are more frequently used to assess implant stability [6,7]. One of the most common tools for assessing primary stability is Periotest (Medzintechnik Gulden e K, Modautal, Germany). A small pistil built in this device is accelerated toward the target (tooth, implant), which is deflected depending on its periodontal/peri-implant status [10]. A digital range that varies from −8 to +50 was used to assess the damping properties of the peri-implant tissue, where the lower values show greater implant stability [11].

A method that can be applied to ameliorate the process of bone healing and to increase the stability of implants is a low-level laser therapy (LLLT) [12]. The LLLT or another term PBM (photo biomodulation) induces a nonthermal photochemistry effect on the cellular level following an increase of ATP production in mitochondria. [13]. Various research proved an improvement in the stability of implants and the bone to implant contact (BIC) factor, after near-infrared laser application [6,14,15]. PBM at low-energy doses excites the mitochondrial and cellular photoreceptors to synthesize ATP, which enhances cell proliferation rate [16]. Moreover, PBM enhances the proliferation and differentiation of osteoblasts [17,18]. In their studies, AlGhamdi et al. [16] unveiled that PBM can induce mitosis in cultured cells, production of collagen, and DNA and RNA synthesis. Furthermore, Mohammed et al. [19], in their investigation, confirmed that the LLLT reinforces the revitalization process, improves nerve regeneration, and the healing of damaged tissues.

To date, there has been minimal research investigating the effect of PBM on orthodontic mini-implants’ stability [20,21]. However, no randomized split-mouth controlled trial before has evaluated the role of low-level laser energy on mini-implants stability in orthodontics. Thus, the study aimed to estimate clinically an influence of a 635 nm diode laser on the stability of orthodontic mini-implants placed in a maxilla. Furthermore, mini-implants’ failure rate (mini-implant loss) and a pain level after the treatment was evaluated.

## 2. Materials and Methods

The trial was designed as a randomized and controlled test. The approval of the Local Ethics Committee of Wrocław Medical University, Faculty of Dentistry was obtained (permission number: KB-278/2018) and an informed consent in accordance with the Helsinki Declaration was obtained from all participating subjects. The clinical trial was registered with ClinicalTrials.gov (identifier: NCT04175405).

### 2.1. Subjects

**Figure 1:**
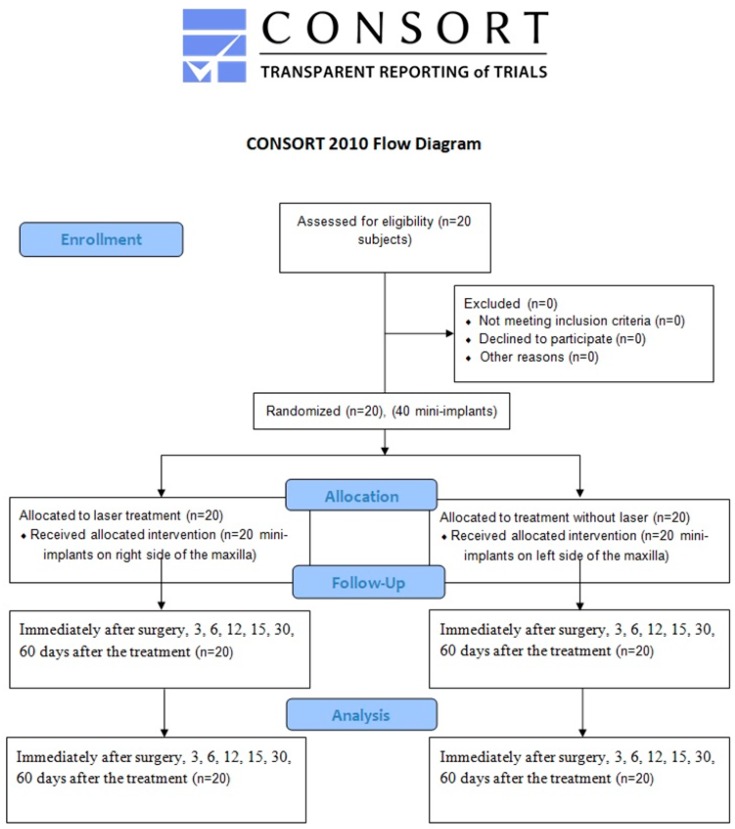
Flowchart of treated subjects according to CONSORT2010.

The study involved an insertion of 40 orthodontic mini-implants in total, in the posterior region of a maxilla in 20 patients (13 women and 7 men; age: 32.5 ± 6.1 years) (Figure 1). Sample size was calculated to be 20 in each group (side of the maxilla) using G×Power (Kiel University, Kiel, Germany) software assuming 80% power of study, 95% confidence interval, level of significance of 0.05, and *d* = 0.58 [12]. All of the patients were treated by the same implantologist. The subjects were chosen for the study under the following inclusion criteria: patients with a class II malocclusion defect requiring lateral maxillary teeth distalization based on the use of an orthodontic mini-implant; all of the patients were treated for the first time using fixed orthodontic appliance; no systemic diseases; were not using anti-inflammatory drugs; had not used antibiotics in the previous 24 months; were non-smokers; had no uncompensated diabetes or uncontrolled periodontal disease; no history of radiotherapy, or taking bisphosphonate medication; and each patient has undergone hygienist treatment before the clinical trial.

### 2.2. Orthodontic Treatment

Patients qualified for the study were treated with a straight wire technique using fixed appliances with MBT .022″ prescription (GC Orthodontics America Inc., Alsip, IL, USA).

### 2.3. Surgical Procedures

**Figure 2:**
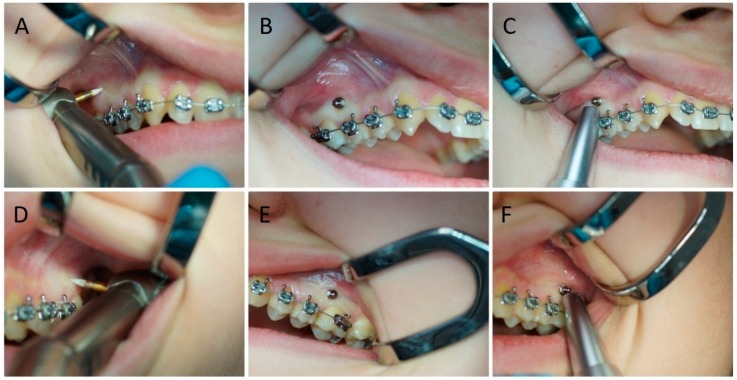
The orthodontic mini-implants insertion procedure on the left and right side of the maxilla. (**A**,**D**) Removal of soft tissue with a ceramic bur. (**B**,**E**) Mini-implant insertion. (**C**,**F**) Control of mini-implant stability by using a Periotest device.

A total number of 40 orthodontic mini-implants (RMO, West Colfax Ave., Denver, CO, USA), made of titanium alloy (grade 5), 10 mm long with a diameter of 1.4 mm, were placed in the area of the attached gingiva between second premolar and first molar teeth 2 mm below mucogingival junction of both sides of maxilla. The soft tissue was removed using a ceramic bur, and each implant was placed immediately with a hand driver without bone decortication. (Figure 2) After the procedure, additional mouthwash was prescribed; 10 mL of 0.1% chlorhexidine mouthrinse (Eludril, Pierre Fabre, France) for 60 s 3 times a day for 2 weeks.

### 2.4. Laser Application

**Figure 3:**
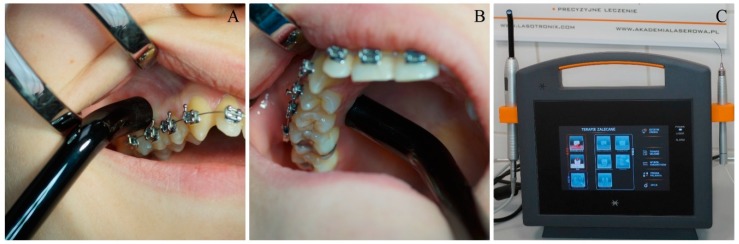
PBM with 635-nm diode laser on (**A**) a buccal and (**B**) a palatal side of the alveolus/implant. (**C**) Diode laser SmartM (Lasotronix, Poland) used in the study.

The coin toss was done to choose the side of the laser application in the maxilla. In our study we applied a red diode laser (SmartM, Lasotronix, Piaseczno, Poland) at 635-nm wavelength at the following set parameters; output power: 100 mW, handpiece diameter: 8 mm, spot area: 0.5024 cm^2^, average power density: 199.04 mW/cm^2^, continuous mode, dose: 10 J per point (20 J/cm^2^), time: 100 s per point, 2 points (irradiation on a buccal and a palatal side of the alveolus/implant), total energy per session 20 J. The diode laser was used in contact mode with peri-implant soft tissue on the right side of the maxilla (group G1) according to the following irradiation protocol: immediately and 3, 6, 9, 12, 15, 30 days after surgery. The total energy after all therapeutic sessions was 140 J. (Figure 3) The subjects with the implants inserted on the opposite (left) side of the maxilla were a control group (G2).

### 2.5. Measurement of Implants’ Stability

The mini-implants’ stability was estimated employing a Periotest device (Medzintechnik Gulden e K, Modautal, Germany). The Periotest measurement system includes the sound formed from contact between an object and a metallic tapping bar in a handpiece, which is electromagnetically driven and electronically verified. The Periotest response detection is analyzed through a fast Fourier transform (FFT) algorithm. Simply put, the Periotest answer to tapping is estimated by an accelerometer and then analyzed. The signal generated by tapping is then transformed to a value called the Periotest value (PTV), which depends on the damping characteristics of the peri-implant tissue [21]. The Periotest Test values (PTVs) are based on a numerical scale ranging from −8 to +50, defined by a mathematical computation. The lower Periotest values indicate higher implant stability and thereupon the higher absorption effect of the target objects. Calculation of mini-implants stability in the study was conducted: immediately and 3, 6, 9, 12, 15, 30, and 60 days after the insertion. In each follow-up period, the measurements were done 5 times, and mean results were assessed and compared.

### 2.6. Measurement of Pain Value

**Figure 4:**
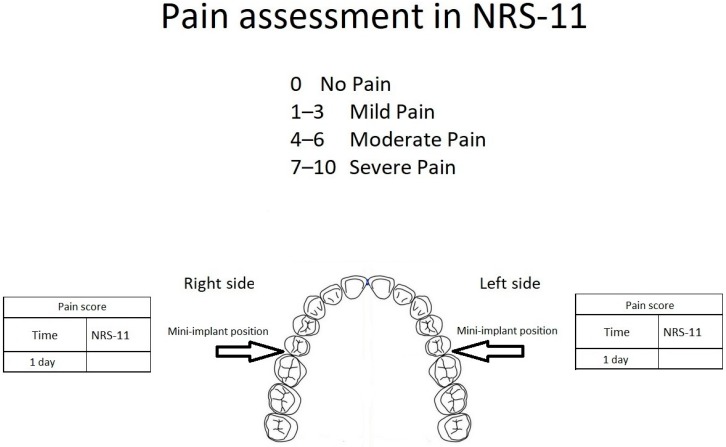
Pain assessment on the NRS-11 scale on both sides of the maxilla during the first day after the treatment.

Immediately after the mini-implants’ placement, each patient received a questionnaire for individual pain assessment (the numeric rating scale, NRS-11, grade level 0–10). The maximum pain level was measured on both sides of the maxilla during the first day after the treatment. The NRS-11 scale consists of a conscious, subjective assessment of the pain experienced; therefore, it is used in the case of patients over ten years old. A rating of 0 signifies no pain, 1–3 represents mild pain, 4–6 moderate pain, and 7–10 severe pain (Figure 4).

### 2.7. Statistical Analysis

To assess whether the data were normally distributed, the Kolmogorov–Smirnov test was performed at the 95% level. Differences in mini-implants’ stability and pain value were compared with the dependent sample Student’s *t*-test using the Statistica 12 software (version, StatSoft, Krakow, Poland) at a significance level of *p* = 0.05.

## 3. Results

We found significantly higher secondary stability, lower mean PTV (6.18 ± 5.30) and (1.51 ± 2.25), for self-drilling mini-implants (G1, test group) in contrast to the control, G2 group (9.17 ± 8.25) and (5.00 ± 3.24), after 30 (*p* = 0.0003) and 60 days. (*p* = 0.0000) (Table 1).

Moreover, the analysis of the mini-implant stability after a 635-nm diode laser application revealed significant higher stability for the test group (PTV–2.61 ± 0.47) in comparison with no irradiated mini-implants (PTV–1.05 ± 1.13) after three days (*p* = 0.0000) (Table 1).

**Figure 5:**
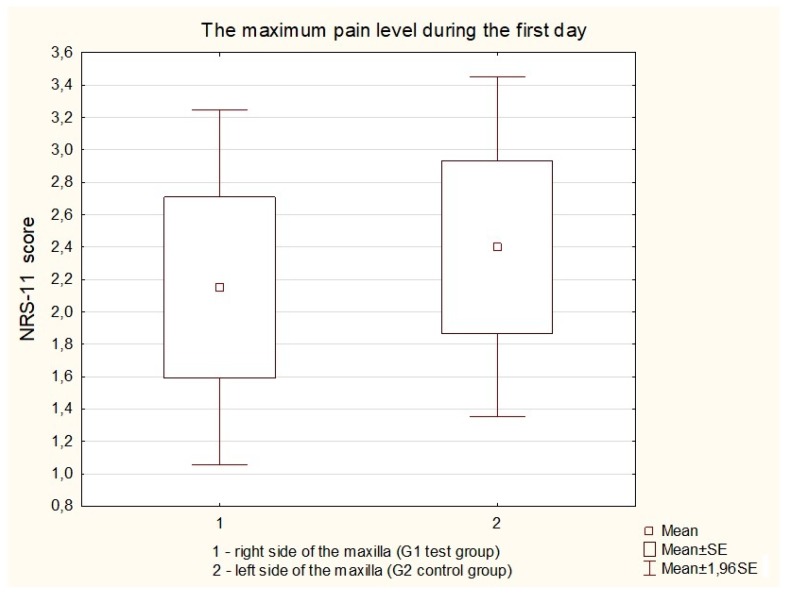
The maximum pain level during the first day after mini-implant insertion measured on the NRS-11 scale.

Furthermore, there was no significant difference in pain level measured on the NRS-11 scale on both sides of the maxilla. (*p* = 0.3665) (Figure 5).

An important finding was that all inserted mini-implants survived during the two months observation period.

## 4. Discussion

The PBM is a minimal-invasive treatment allowing to stimulate cellular processes such as ATP, DNA, and RNA synthesis [16,22]. Many authors demonstrated the positive effect of LLLT in bone healing [22], osteoblasts [22], fibroblasts [13] proliferation, nerve repair [19], increased peri-implant bone density [23], and reduced pain in orthodontics [24]. Our study aimed at testing the photomodulation effect of the LLLT on implant stability of orthodontic mini-implants placed in a maxilla and mini-implants failure rate and a pain level after the treatment. The main finding of the study was that the mini-implants irradiated with a 635-nm diode laser at 20 J/cm^2^ accounted to significantly greater secondary stability (after three days, one and two months) in contrast to non-irradiated side. Furthermore, a similar score of pain level was recorded on both sides of the maxilla, which agrees with findings of other randomized clinical split-mouth study concerning the effect of the application of low-level laser with 4-Joule or 16-Joule energy on pain reduction following elastomeric separators placement [9].

Photobiomodulation therapy in a wavelength spectrum of 600–1100 nm (optical window) appears in a more extensive penetration depth what causes a more significant cell-light answer [23]. LLLT effect depends on the energy dose, which was described by Arndt-Schultz’s curve. Arndt-Schultz’s curve implies that a low stimulus improves physiologic activity, moderate stimuli hinder the activity, and strong stimuli eliminate the activity [22,23]. That suggests that the application of PBM at a too low dose has no biological effect. However, if too high energy is used, a bio-suppressive effect will occur. The near-infrared laser light application at energy density (fluence) in the range of 1–10 J/cm^2^ is optimal to obtain an optimal biological answer [23]. In our present study, a dose per point of 10 J (20 J/cm^2^) allowed increasing the secondary mini-implant stability.

The interesting findings of our present study was a slower trend of reduction in implant stability for irradiated mini-implants during the first two weeks as compared to the control group. This phenomenon could be explained by the effect of the red wavelength on the inflammation phase of the bone after the injury evoked by placing the implants [23]. In the peri-implant area, several changes can be observed [25]. After the first 2 h up to the end of the third-day following processes occurring, e.g., blood clot and granulation tissue formation, and development of provisional matrix rich in vessels, mesenchymal cells, and fibers [25]. Next, the provisional connective tissue is formed. This process is described as fibroplasia and angiogenesis and lasting from fourth to the seventh day. In the second week of the inflammation phase, a formation of new woven bone is observed. That is the first phase of the osseointegration process [25]. The infrared laser can change, ameliorate, and improved each process occurring in the first inflammation phase [23]. That was a reason why the 635-nm diode laser was used five times during the first two weeks after the mini-implants insertion in our study.

To date, no randomized split-mouth controlled trial before in humans has evaluated the role of low-level laser energy on mini-implants stability in orthodontics. However a few studies concerning the use of LLLT on orthodontic mini-implants stability were commenced. [20,21] In the study of Omasa et al. [20] the authors found a significantly lower Periotest value (0.79- to 0.65-fold) and the higher newly formed bone volume(1.53-fold) in the LLLT (830 nm, continuous emission, 200 mW, 195 J/cm^2^, 135 sec·2 points; the mesial and distal sides of the mini-implant, 54 J per session) group in contact to non-irradiated mini-implants in a rat model. It should be highlighted that in the study of Omsa et al. the authors used higher energy dose per session in contrast to our present study and out of the optical window range (1–10 J/cm^2^) described by Arndt-Schulz curve. The positive results of PBM in mini-implants stability were confirmed also by the study published by Pinto et al. [21] in the rabbit model. The authors proved that the diode laser with a wavelength of 808-nm (energy of 2.5 J, 2 points, 5 J per session, 10 sessions) increased the mean pull-out force values in contrast to control group. The energy dose per session was lower in contrast to this used in our study; however, Pinto et al. applied the diode laser with an 808-nm wavelength which has greater penetration depth than a 635-nm diode laser.

The second aim of our study was to determine the effect of the LLLT on pain level in the same day when placed orthodontic mini-implants. In our study there was no significant difference in pain level measured on the NRS-11 scale on both sides of the maxilla. In the scientific literature, there is no other evidence proving our findings. However, in the split-mouth clinical study presented by AlSayed et al., the authors obtained no difference in relieving elastomeric separators induced orthodontic pain after LLLT, applied at two different laser energy values (4 and 16 J). However, there is some bias in pain analysis in this study because the pain feeling by the patients could alsom be caused by the fixed orthodontic appliance [24]. There is no consistent thesis on pain during orthodontic treatment. Earlier investigations by Luppanapornlarp et al. [26] recorded that higher forces applied to the teeth were correlated with more severe pain. Then, the authors compared the level of the orthodontic pain correlated with exerting different forces using nickel-titanium coil springs on segmented archwires. An increase in the adjusted force results in a corresponding rise in the deformation of orthodontic wire. That feature of the material is known as elastic straining and influences the properties of orthodontic wire. Discomfort and pain usually begin 2 to 4 h after the fixed orthodontic appliance placement (application of force) and progress for 24 h and progressively disappear in the next seven days [24,27].

Furthermore, the mini-implants’ failure rate (mini-implant loss) for a 60-day observation period was evaluated. In our present study, all thirty implants survived and serve as an anchorage system in the orthodontic treatment. Our previous in vitro study [5] in mandible showed that insertion of the mini-implants without bone decortication could be conducted without the implant fracture. In that previously conducted in vitro experiment, we concluded that the high initial stability and low diameter of the mini-implant effects result in an increased risk of breakage, especially for the self-drilling system [5]. Hence, we recommend perforating the cortical lamina of the bone with a density of over 840 HU to reduce the forces needed to insert implants with minimal PTV values needed to anchor the orthodontic forces. In the present study, all of the mini-implants were inserted without bone decortication in the maxilla. The thickness of the cortical lamina in the maxilla is lower in contrast to the mandibular arch. Bone decortication was not performed to achieve higher initial stability of the mini-implants. The smaller cortical lamina thickness and bone density of the maxillary arch reduce the risk of the fracture of a thin mini-implants inserted by using the self-drilling method [5,28].

Necessary attention should be paid to preventing the thermal injury of the periodontal tissues when irradiating tissues with lasers [29,30,31,32,33]. A temperature increase by 10 °C for 60 s on the outer root surfaces can cause irreversible damage to the periodontal ligament and bone, leading to bone resorption and tooth ankylosis [29,30,31]. A tissue temperature gradient (∆Ta) below 10 °C should be regarded as optimal and harmless [29,30,31,32,33,34]. To guarantee the safeness of the irradiated tissues as well as determine the predictability of treatment results, it is predominant to set the proper lasing parameters and treatment guidelines [29,30,31,32,33,34,35]. The literature proved the safeness of various laser systems applied in implant dentistry. Matys et al. [32] obtained the thermal rise of implants below critical 10 °C for Er:YAG laser (2 W and 30 s exposure time) and a diode laser with a wavelength of 980-nm (2 W, 30 s exposure time, 60 J). It was proved in the literature that LLLT application using doses recommended in the World Association for Laser Therapy (WALT) guidelines (dosage 1–4 J per point) does not cause a high thermal increase of irradiated tissues [19,35]. However, in this study, we used the 635-nm diode laser (100 mW, exposure time of 100 s per point) at an energy dose of 10 J, which is higher than that recommended by WALT. Notwithstanding, the study of Joensen et al. [35] showed insignificant (5.5 °C) thermal increase in light and medium skin for 200 mW, 810 nm laser, and a 60 mW, 904 nm diode laser at 9 and 12 Joules. Thus, the dose of 10 J that was used in our research seems to also be safe for the peri-implant area.

Additional studies using PBM and mini-implants applied in orthodontics are required to determine long-term clinical benefit in the described method. To evaluate the influence of the LLLT (red and infrared wavelengths) on peri-implant bone tissue, additional randomized-controlled trials in the long-term and on larger study groups are recommended.

## 5. Conclusions

Irradiation of peri-implant soft tissue using a 635-nm diode laser enhances secondary mini-implant stability after three days, one month, and two months. The diode laser application has no significant effect on pain level after orthodontic appliance placement measured in the NRS-11.

## Figures and Tables

**Table 1:** Results of Periotest value (PTV) of mini-implants at different time points on the test and control side.

Period	Laser	Std	Control	Std	df	*p*-Value
Baseline	−2.74	0.70	−2.53	0.58	19	0.1231
3 days	−2.61	0.47	−1.05	1.13	19	0.0000
6 days	−2.71	0.12	−2.60	2.79	19	0.8644
9 days	−1.14	0.27	−0.16	4.04	19	0.3212
12 days	−0.08	0.95	1.62	5.49	19	0.1590
15 days	0.65	1.55	3.18	6.78	19	0.0582
30 days	6.18	5.30	9.17	8.25	19	0.0003
60 days	1.51	2.25	5.00	3.24	19	0.0000

Std—standard deviation, df—degree of freedom.
